# Anti-RSV Peptide-Loaded Liposomes for the Inhibition of Respiratory Syncytial Virus †

**DOI:** 10.3390/bioengineering5020037

**Published:** 2018-05-09

**Authors:** Sameer Joshi, Atul A. Chaudhari, Vida Dennis, Daniel J. Kirby, Yvonne Perrie, Shree Ram Singh

**Affiliations:** 1 Center for NanoBiotechnology Research, Alabama State University, Montgomery, AL 36016, USA; sjoshi@alasu.edu (S.J.); atulvet@gmail.com (A.A.C.); vdennis@alasu.edu (V.D.); 2 Aston Pharmacy School, Life and Health Sciences, Aston University, Birmingham B4 7ET, UK; d.j.kirby1@aston.ac.uk; 3 Strathclyde Institute of Pharmacy and Biomedical Sciences, University of Strathclyde, 161 Cathedral Street, Glasgow G4 0RE, UK; yvonne.perrie@strath.ac.uk

**Keywords:** liposomes, respiratory syncytial virus, peptide, hydrophilic

## Abstract

Although respiratory syncytial virus (RSV) is one of the leading causes of acute respiratory tract infection in infants and adults, effective treatment options remain limited. To circumvent this issue, there is a novel approach, namely, the development of multifunctional liposomes for the delivery of anti RSV-peptides. While most of the peptides that are used for loading with the particulate delivery systems are the penetrating peptides, an alternative approach is the development of liposome-peptide systems, which are loaded with an RSV fusion peptide (RF-482), which has been designed to inhibit the RSV fusion and block infection. The results of this work have revealed that the liposomes themselves can serve as potential RSV inhibitors, whilst the anti-RSV-peptide with liposomes can significantly increase the RSV inhibition when compared with the anti-RSV peptide alone.

## 1. Introduction

Respiratory syncytial virus (RSV), as well as Rhinovirus (HRV), are the primary causes of acute lower respiratory tract (LRTI) infections [1]. An RSV infection is particularly noticeable during winter for populations, including the fetus, infants [2], young children, and adults [3,4,5]. A focused cause of bronchitis and pneumonia is RSV. It is widely recognized that there is a need for a vaccine against RSV, as the natural infection is not capable of providing life-long immunity and patients are prone to suffer from repeated RSV infection [6].

RSV, which belongs to the *Pneumoviridae* family, is a distinct serotype that has two major antigenic circulating subgroups (A & B), of which the A is dominant [5,7,8]. RSV has an RNA genome consisting of 15,191 base pairs. The virus can be identified with 11 proteins, including 2-non-structural proteins (NS-1 and NS-2), 3-surface proteins (glycoprotein-G, fusion protein-F, and small hydrophobic protein-HP), two overlapping frames of M2 mRNA producing 2 distinct transcription factors (M-1 and M-2), and four other structural proteins (matrix protein-M, nucleocapsid-N, phosphoprotein-P, and large protein-L) [5]. The viruses of the *Pneumoviridae* family fuse their membrane with the plasma membrane of the host, which results in a cell fusion if it is added to the cell in large quantity [9]. The entry of the RSV virus into the host cells occurs with the aid of the fusion protein-F, which has two hepated-repeated regions that form a hairpin-like structure, which facilitates the entrance of the virus into the cells [10].

The root cause analysis of any disease or disorder is at the foundation of the treatment design. An infection of RSV can start with a mild upper respiratory tract infection (URTI) and may lead to a potentially precarious lower respiratory tract infection [5]. RSV transmission occurs from person to person contact, directly or indirectly; an RSV infected person, upon sneezing or coughing, can leave viral droplets suspended in the air, which have the potential for transmission of the infection by entering the healthy individual through the mouth, nose, or eyes [11].

The first line treatment of RSV infection is the use of bronchodilators, such as α and/or β adrenergic agonist [5]. For pediatrics, since corticosteroids are not approved for treating RSV-infected individuals that are less than 1 year old, because of safety concerns [12], the use of vaporub and non-aspirin formulations, such as paracetamol, are the treatments of choice prior to clinical attention. Of the very few options available for the treatment of RSV, Ribavirin, a broad spectrum antiviral drug, is used. Although this too comes with limitations and drawbacks [13]. Despite several concept studies that have claimed the effectiveness of ribavirin in significantly reducing the RSV load and minimizing the disease severity, the disadvantages of the mutagenicity, teratogenicity, and carcinogenicity have subsequently resulted in FDA denial [14]. An active rather than passive prophylaxis would be a better choice but, unfortunately, there is no current vaccine that has been developed for the RSV infection. The formalin-inactivated vaccine was launched in the 1960s but was later withdrawn because of inadequate immunogenic responses, as well as an atypical T_H_2-type response, which increases the chances of reinfection with similar or deadly infections [12].

The use of nanoparticulate systems, such as liposomes, can provide adjuvant action by an enhanced antigen delivery or by inducing an innate immune response [15]. Commercially, liposomes are the most successful carrier systems across the globe. Liposomes are vesicles with an aqueous core, which are surrounded by a phospholipid bilayer. From the numerous pre-clinical and clinical studies, it is clear that those liposomes are not only carriers of the chemical and biological materials, but they are also non-toxic and good at retaining efficacy. Indeed, the potential of the liposomes to act as a latent carrier of active pharmacological agents is well established [16,17,18], whilst liposomes have also shown their ability to carry antigens and to serve as immunomodulators [19]. Recently, a novel approach to inhibit the RSV has been the use of gold nanoparticles (GNPs) [20]. These GNPs can be functionalized with nucleic acid, antibodies, drugs, and peptides, and these functionalized GNPs then can be applied in the diagnosis or treatment of the diseases [21]. The anti-RSV fusion peptide RF-482 (sequence: VFPSDEFDASISQVNEKINQSLAFIRKSDLLHNVNAGKK) is used to functionalize the GNPs, and a significant inhibition of the RSV was observed after the loading of the GNPs [20]. The liposomes can be loaded with peptides, however insufficient research has been performed using liposomes as carrier systems for the inhibition of RSV [22,23,24]. Therefore, considering the global need and structural attributes of liposomes, this work describes an approach that employs the liposomes for the loading of RF-482, for the enhanced inhibition of RSV.

## 2. Materials and Methods

### 2.1. Materials

The lipid 1,2-disteroylphosphatidylcholine (DSPC) was purchased from Avanti Polar Lipids, Inc. (Alabaster, AL, USA). The cholesterol was obtained from Sigma-Aldrich Co. (St Louis, MO, USA). The minimal essential medium (MEM), Dulbecco’s Modified Eagle’s media (DMEM), Hank’s balanced salt solution (HBSS), fetal bovine serum (FBS), 7-amino actinomycin D (7-AAD), l-glutamine (100 mM), antibiotics, TrypLE™, Lipofectamine 2000, real-time probe, primers, SuperScript^®^ II Reverse Transcriptase, and TaqMan^®^ Master Mix 2× were purchased from Life Technologies (Thermo Fisher Scientific, Waltham, MA, USA). Both the DNA and ribonucleic acid (RNA) isolation kits were purchased from Qiagen (Germantown, MD, USA). The human epithelial type 2 (HEp-2) cells were obtained from American Type Culture Collection (ATCC^®^, Manassas, VA, USA). The cell toxicity was analyzed using the 3-(4,5-dimethylthiazol-2-yl)-2,5-diphenyltetrazolium bromide (MTT) dye-based cell proliferation assay kit from Promega Corp (Madison, WI, USA). The osmium tetroxide (4% solution) that was used for the fixative staining of the liposomes was purchased from Electron Microscopy Sciences (Hatfield, PA, USA). The ethanol was purchased from Fisher Scientific, Fair Lawn, NJ, USA. All of the reagents that were used for the experiments were of analytical grade.

### 2.2. Preparation of Small Unilamellar Liposomes

The liposomes were prepared using the thin-film hydration method [25]. Briefly, the lipid DSPC and cholesterol (5:2 *w*/*w*) were dissolved in an organic solvent mixture of chloroform and methanol, with the addition of the lipophilic drug, followed by a solvent evaporation to obtain a thin, dry film. The film was then hydrated with a phosphate buffered saline (PBS) (1 mM, pH 7.4, 25 °C), which resulted in the formation of the multi-lamellar vesicles (MLV). This MLV suspension was then sonicated using a probe sonication so as to obtain small unilamellar vesicles (SUV). The sonicated liposomal suspension was then centrifuged to remove titanium debris.

### 2.3. Loading of Peptide

The liposomal suspension was obtained after the removal of the titanium debris was used for the peptide loading. The RF-482 (500 µg) was added to the liposomal suspension (2.0 mL) and it was subjected to the mechanical shaking for 30 min. The non-associated peptide was then removed, using centrifugal filter units (Ultracel-50K, Millipore Ireland Ltd., Cork, UK). The RF-482 loaded liposomes were then used for further studies.

### 2.4. Particle Characteristics

Dynamic light scattering (DLS) (Zetasizer Nano-S, Malvern Instruments, Westborough, MA, USA) was used for the size determination of the empty and RF-482 loaded liposomes. The zeta potential was determined using the laser Doppler velocimetry (Zetasizer Nano-S, Malvern Instruments, Westborough, MA, USA). The samples were prepared using the PBS that was diluted 1–300 times (pH 7.4, 25 °C).

### 2.5. Transmission Electron Microscopy (TEM)

Osmium tetroxide has commonly been used for the fixative stain for cells in the TEM analysis. Since the liposome structure resembles cell structure, a similar fixative staining was performed in this study. Briefly, a drop of each liposome sample was placed on to the carbon film mesh copper grid, and the excess suspension was removed using a filter paper. The staining was performed using a 4% osmium tetroxide solution and the images were captured using a high-resolution TEM (EM10A/B, ZEISS, Oberkochen, Germany).

### 2.6. Quantification of Peptide Association

After the separation of the associated and non-associated peptide, the eluent and the liposome suspension were tested for peptide presence using the micro bicinchoninic acid (BCA) assay kit (Thermo Scientific, Rockford, IL, USA), according to the manufacturer’s protocol. Briefly, the standard curves were prepared using the RF-482 peptide, for linearity and calibration. All of the samples were diluted using PBS, and since ethanol was used to separate the liposome associated protein, this was also further diluted with PBS. The standards and samples (150 µL each) were each added to wells of a 96-well plate, which was followed by the addition of 150 µL of working reagent and incubation for 2 h at 37 °C. The plate was then cooled to room temperature, and the absorbance measurements were taken at 562 nm, using an ELISA plate reader (TECAN^TM^, Morrisville, NC, USA). Apart from the standards and samples, the ethanol, buffer, and empty liposomes were tested for BCA interference.

### 2.7. Cell Viability Assay

The CellTiter 96^®^ Non-Radioactive cell proliferation assay kit (Promega) and the MTT (3-(4,5-dimethyl-thiazol-2-yl)-2,5-diphenyl-tetrazolium bromide) dye was used to assess the cell toxicity of the empty and peptide-loaded liposomes, as well as the peptide RF-482 to human HEp-2 cells. The human HEp-2 cells were propagated using MEM, which was supplemented with 10% FBS, 2 mM l-glutamine, 75 U/mL penicillin, 100 mg/mL kanamycin, and 75 mg/mL streptomycin (MEM-10). Each well of a 96-well plate was seeded with 25,000 cells and incubated overnight at 37 °C in a 5% CO_2_ atmosphere for adherence. Two different concentrations of the peptide RF-482 (0.01 mg and 0.02 mg), empty liposomes and peptide-loaded liposomes were added to the wells, and the cell toxicity was assessed 72 h post incubation. The absorbance was measured at 570 nm, using an ELISA plate reader (TECAN^TM^). The percent viability was then calculated from a comparison of the samples against the negative control.

### 2.8. Fluorescence Microscopy

The fluorescein isothiocyanate (FITC) labeled peptide RF-482 was used to demonstrate the association of the peptide with the liposomes. However, in order to study the RSV inhibition, 30,000 cells per well were seeded into an 8-chambered slide. The cells were incubated with peptide RF-482, empty liposomes, and peptide-loaded liposomes for 48 h, followed by fixing it in a paraformaldehyde-glutaraldehyde and buffer (PBS) wash. The nuclei were stained using DAPI and the cell membranes were stained using Cell Mask™ (Life Technologies). All of the chamber slides were imaged using the DAPI and FITC channels of the Nikon Ti Eclipse fluorescence microscope (Nikon Inc. Melville, NY, USA).

### 2.9. Plaque Assay

The plaque assay was one of the most common and reliable methods for the determination of the viral/antiviral activity in the cell cultures. The plaque assay was performed using the HEp-2 cells (1.5 × 10^5^/well) that were cultured in MEM-10 for 48 h, in order to achieve a maximum confluency. Dilutions of a mixture of RSV and peptide, empty liposome, and peptide-loaded liposomes were prepared in DMEM before the infection. Post-infection, the cells were covered by immobilizing the overlaying medium (1.6% Methylcellulose) and were subsequently incubated for five days at 37 °C in 5%, CO_2_ environment. On day 5, the overlaying medium was removed, and the monolayer was fixed with cold methanol at −20 °C, followed by being stained with a 0.1% crystal violet solution. The plaques were counted in order to determine the viral or antiviral activity.

### 2.10. TaqMan qPCR Analysis

The HEp-2 cells (1.5 × 10^5^/well) were seeded in 12-well plates, followed by treatment with varied RSV dilutions (10^2^ to 10^8^), with and without the peptide, empty liposomes, or peptide-loaded liposomes. These treated cells were then incubated for 48 h at 37 °C in a 5% CO_2_ environment, which was followed by being harvested for RNA extractions. A total of 1 μg RNA/sample was converted to cDNA, using reverse transcriptase according to the manufacturer’s protocols (Applied Biosystems, Waltham, MA, USA). TaqMan qPCR was performed for the amplification of the RSV-F gene, using the RSV-F gene-specific primers and probe, and previously published procedures [26,27]. The RSV-F gene amplicon dilutions (10^2^ to 10^8^) were used as the standards. The qPCR for each sample was run in a duplicate on the Applied Biosystems^®^ ViiA™ 7 real-time PCR (Life Technologies). The fold changes were calculated by comparing the untreated cells.

### 2.11. Statistical Analysis

Unless stated otherwise, the results were calculated as the mean ± standard deviation (SD). The data were analyzed by the student’s *t*-test, alone, or by ANOVA, which was followed by Dunnett’s post-hoc analysis for comparison. The significance was acknowledged for *p* values < 0.05.

## 3. Results

### 3.1. Confirmation of Peptide Loading

**Figure 1:**
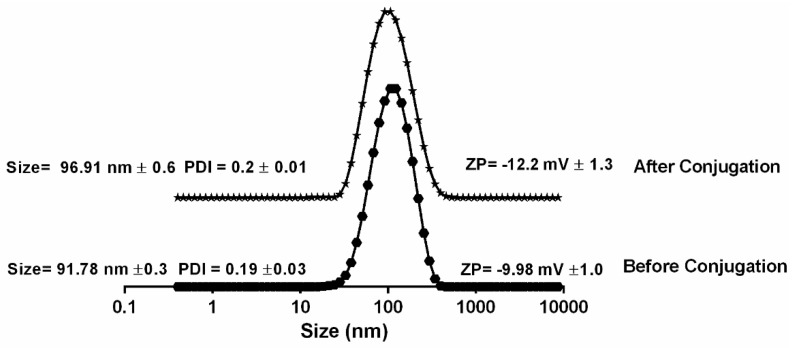
Dynamic light scattering (DLS) measurement of liposomes before and after RF-482 loading. (*n* = 3 ± SD).

Liposomes are spherical vesicles made up of phospholipids [25]. Upon the hydration of a thin lipid film, MLVs were formed by vortexing and heating the suspension above the transition temperature (Tc) of the lipid. These MLVs could then be transformed into SUV by a variety of methods, including a probe sonication, which was used in this study. The liposomes were one of the most flexible structures that conjugate moieties, like lactose and peptide [22,28]. There was a slight change in the size of the liposome that was observed after the loading with RF-482 (Figure 1).

**Figure 2:**
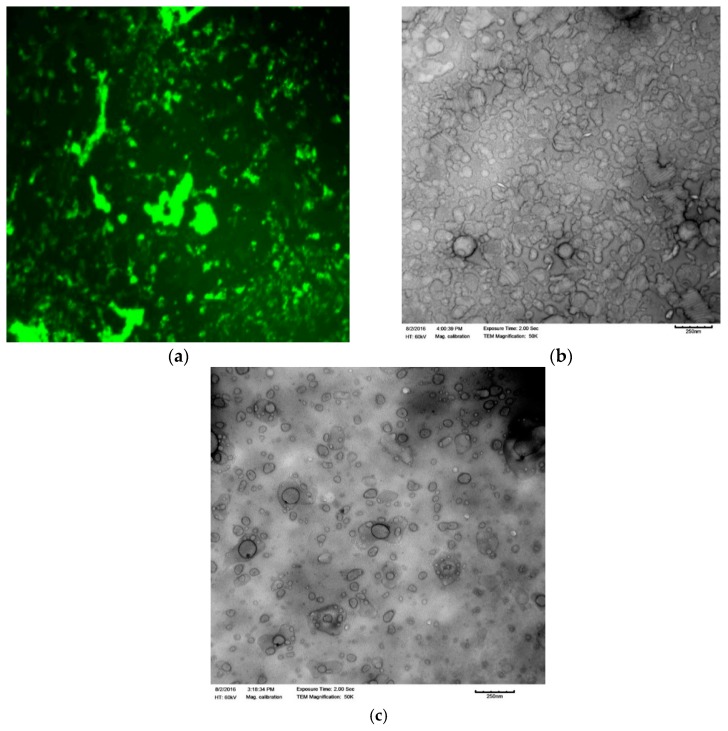
Fluorescence microscopy analysis of the presence of the fluorescein isothiocyanate (FITC)-labeled peptide RF-482 (green) confirms association with liposomes (40× magnification) (**a**). Transmission electron microscopy analysis. Comparison between the empty liposomes (**c**) and the RF-482 peptide-loaded liposomes (**b**).

The average hydrodynamic size, before and after the liposome loading, was 91.78 nm ± 0.3 (PDI 0.2 ± 0.01) and 96.91 nm ± 0.6 (PDI 0.19 ± 0.03), respectively. However, no change was observed on the zeta potential of the liposomes before and after the peptide loading. The first confirmation of the association of the peptide with the liposome was observed when the FITC-labeled peptide RF-482 was used for the loading. After the separation of the unloaded peptide, the liposomal suspension was dried, covered with phosphotungstic acid for better resolution, and observed under the fluorescence microscope (Figure 2a). Subsequently, the empty liposomes, as well as the peptide-loaded liposomes, were imaged under TEM. The presence of protein was confirmed by a cloudy environment that was observed around the peptide-loaded liposomes (Figure 2b). This was not observed in the case of the empty liposomes (Figure 2c).

It was possible that a certain amount of protein might not have crossed the membrane during the separation of the unloaded protein. This non-separated protein, therefore, might get counted as a loaded protein. To eradicate this doubt, three different concentrations of peptide RF-482 were run through the same column for the same amount of time, and the eluents were tested for recovery. It was observed that more than 97% of the protein passed through the column (results not included). This confirmed that the liposomal sample that was obtained after the separation of the unloaded protein would have had negligible amounts of the unloaded protein present along with the liposomes. Therefore, this could be the third confirmation of RF-482 loading with the liposomes.

### 3.2. Quantification of Loading

**Figure 3:**
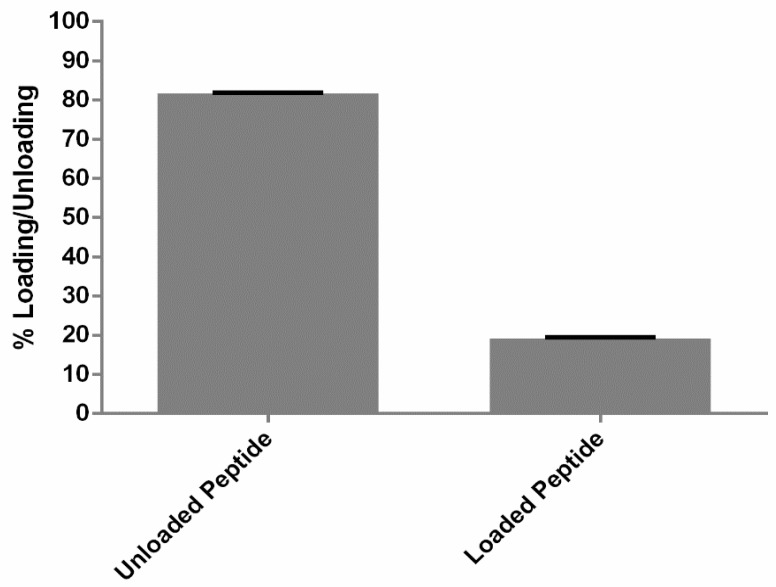
Liposomal loading with peptide RF-482 determined by the bicinchoninic acid (BCA) assay. Results presented as peptide loading determined after separation of the unloaded peptide (*n* = 3 ± standard deviation (SD)).

The exact mechanism of the protein–liposome association is still unknown. However, it was confirmed by the fluorescence microscopy as well as the TEM that the peptide RF-482 was associated with the liposomes. Since the association of the peptide–liposome was confirmed, it was necessary to quantify the amount of protein that was loaded with the liposomes. However, it was equally important to achieve the mass balance so as to study the actual amount of loading of the peptide. Hence, the BCA assay was performed for the analysis of both the eluent and liposome samples. It was confirmed that 81.7% ± 0.1% (*n* = 3) was not loaded and that 19.1% ± 0.4% (*n* = 3) was loaded (Figure 3). This also confirmed the 100% recovery of the initial amount of the peptide.

### 3.3. Cell Toxicity Analysis

**Figure 4:**
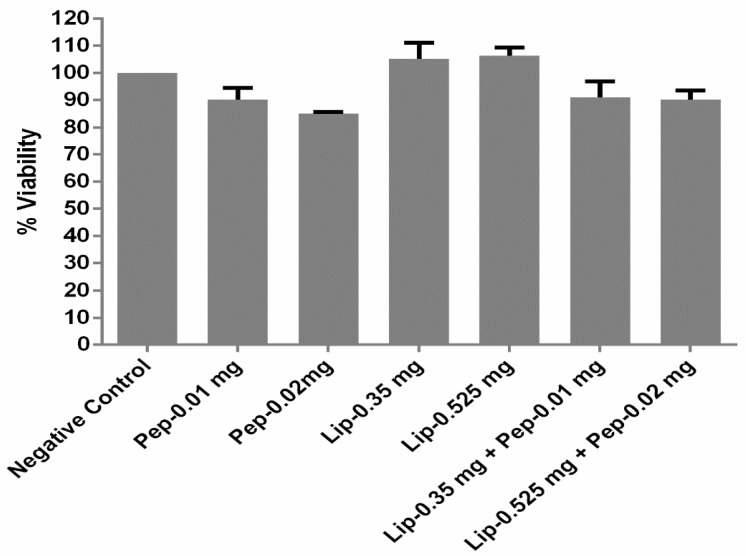
Toxicity profiling of peptide RF-482, liposomes, and RF-482-loaded liposomes presented through cell viability count, performed using the 3-(4,5-dimethylthiazol-2-yl)-2,5-diphenyltetrazolium bromide (MTT) assay (72 h, *n* = 3 ± SD).

The peptide, liposomes, and peptide-loaded liposomes, all at two different concentrations, were tested for their cell toxicity. There was no toxicity observed with both of the concentrations of all of the samples. After 72 h of incubation, the observed cell viability was more than 80% (*n* = 3 ± SD) for the chosen concentrations of the peptide RF-482. Whereas, in the case of the liposome alone, both of the concentrations of lipids were found to be completely non-toxic. However, when the peptides were loaded on to the liposomes, the toxicity was reduced slightly, as the cell viability for the chosen concentrations were observed to be ~90% (*n* = 3 ± SD). (Figure 4).

### 3.4. Evaluation of Viral Inhibition

**Figure 5:**
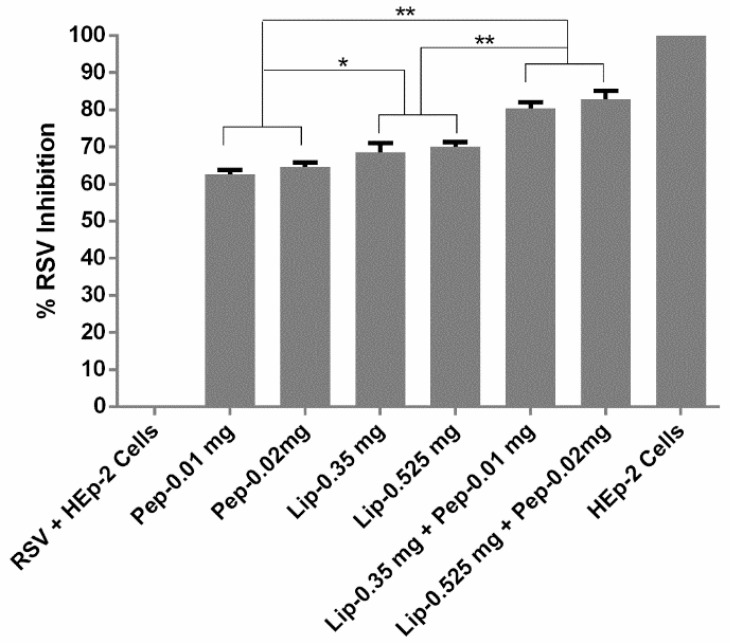
Screening of peptide, liposomes, and peptide encapsulated against respiratory syncytial virus (RSV). Plaques were counted, and the mean plaque was a count of each sample, which was expressed as a percentage of the mean count of the control. (*n* = 3 ± SD). Significant differences between the samples represented as * *p* < 0.05 and ** *p* < 0.01.

The plaque reduction assay has been known as the optimum standard for the antiviral activity analysis [29]. During the plaque assay, the monolayer of the HEp-2 cells was infected with the lytic RSV. The infected cells experienced lytic cycles and eventually appeared as plaques or, in other words, zones of cell death [30]. It was reported recently that the surfactant phospholipids that were bound to the RSV had markedly suppressed the infection though fusion inhibition [31]. We tested the anti-RSV peptide, liposomes, and liposome loaded peptide against RSV infection to HEp-2 cells. When these plaques were counted, it was observed that, although the peptide RF-482 and liposomes alone were capable of inhibiting the RSV, when they were combined, the peptide-loaded liposomes had a significant increase in RSV inhibition (*p* < 0.05, ANOVA, post hoc Dunnett’s multiple comparison tests) (Figure 5). Also, it was an interesting finding that the liposomes alone could also inhibit the RSV, and significantly more so (*p* < 0.05, ANOVA, post hoc Dunnett’s multiple comparison tests) than the peptide RF-482 alone.

**Figure 6:**
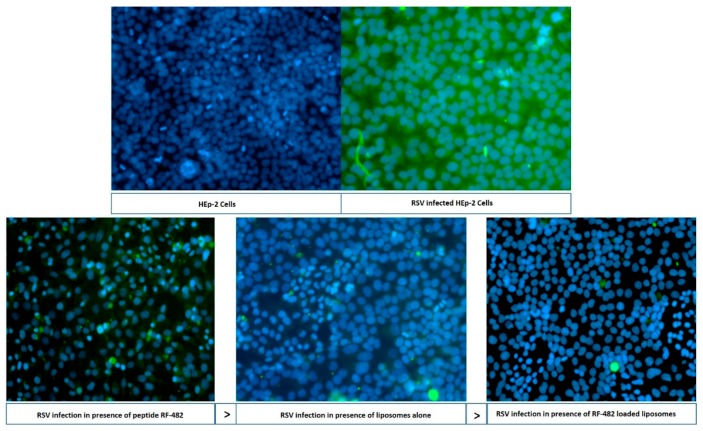
Fluorescence microscopy analysis. FITC (green)—RSV; and DAPI (blue)—HEP-2 cell nucleus. In liposomes and RF-482 liposomes, the blue color represents the survived cells, and the green color represents the presence of RSV.

To validate the plaque assay results, qualitative and quantitative testing was performed. Immunofluorescence imaging was used as a qualitative tool, whilst PCR was employed as a quantitative tool. The cells were incubated for 48 h with the peptide RF-482, empty liposomes, and peptide-loaded liposomes, followed by being fixed in a paraformaldehyde-glutaraldehyde and being washed with a buffer (PBS). An appropriate chamber from the eight-chambered slide was observed under the fluorescence microscope for the RSV activity. The observation that was made from this analysis (Figure 6) was concurrent with the findings that were obtained from the plaque assay (Figure 5) and confirmed that the peptide RF-482, liposomes, and peptide-loaded liposomes, were capable of inhibiting the RSV.

**Figure 7:**
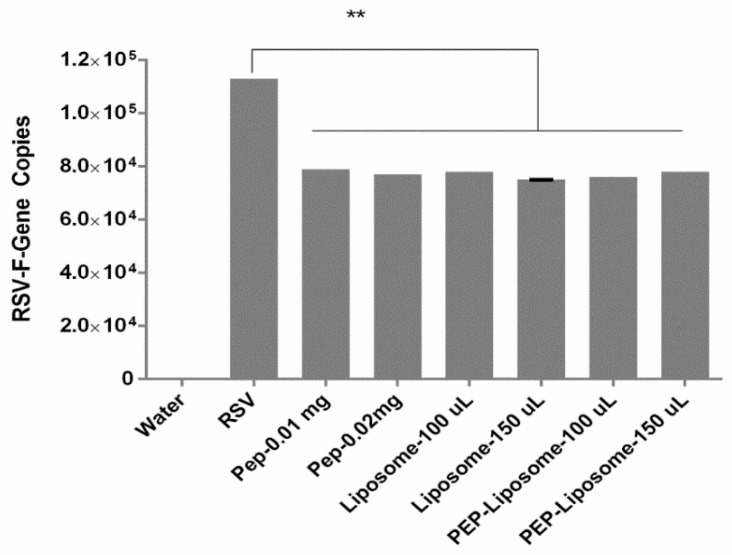
Screening of the RSV-F gene amplicon dilution with water as negative control. Comparison of viral gene amplicon and peptide, liposomes, and peptide-loaded liposomes. (*n* = 3 ± SD). Significant difference between the samples represented as ** *p* < 0.01.

Having the confirmation from the microscopic observation of the viral inhibition, it was necessary to quantitatively validate the plaque assay results. A significant difference (*p* <0.05, ANOVA, post hoc Dunnett’s multiple comparison tests) was observed between a number of gene copies of the virus sample and samples having the peptide, liposomes, or peptide-loaded liposomes (Figure 7). This confirmed that the peptide RF 482, liposomes, and peptide-loaded liposomes were equally capable of inhibiting the RSV. However, although there was no significant difference (*p* >0.05, *t*-test) observed between the peptide, liposome alone, and peptide-loaded liposomes, the loaded liposome samples displayed a trend for slightly lower gene copies, which indicated slightly more inhibition than the individual components when used solely (Figure 7).

## 4. Discussion

To date, the F, G, M, and small hydrophobic proteins have been considered as targets so as to avoid the RSV infection [23,32,33,34]. Nearly a dozen peptide-based formulations have been under clinical trials, and most of these are targeting the F-protein [35,36,37]. However, the role of the F-protein is vital in the spread of the virus, because targeting the G-protein could neutralize the virus, however the actual spread of the virus is only possible after inhibiting the F-protein [38,39,40]. Over a decade ago, studies were suggested that all of the three F-, G-, and RSV-SH protein inhibitors could be used for the complete RSV inhibition [41]. However, within the last decade, several reports indicated that only the F-protein was capable of inhibiting the RSV infection [42,43]. Therefore, some recent studies have specifically targeted the F-protein [20,44,45]. For example, Singh et al. described that the anti-RSV peptide RF-482 was an F-protein inhibitor and was used to functionalize the GNPs, and a significant inhibition was reported after the loading of the GNPs [20].

With the quest of finding an alternative carrier for delivering the anti-RSV peptide RF-482, the liposome formulations were prepared here and were tested for the inhibition of the RSV infection. RF-482 was a small fusion peptide with 39 amino acids, with a total of 611 atoms [20]. Since it was a fusion of the peptide and hydrophilic, it was expected that the RF-482 might have gotten entrapped in the hydrophilic core and that it might have been adsorbed on the surface of the liposomes. Although the exact mechanism of the peptide association with the liposomes was unrevealed, the functionalization of the liposomes was confirmed through the dynamic light scattering (Figure 1), change in the surface charge (Figure 1), fluorescence imaging (Figure 2c), and transmission electron microscopy (Figure 2a,b).

Knowing that the toxicity was an emerging problem in the RSV treatment [7], it was one of the primary objectives when designing the treatment for the RSV infection. The liposomal research to date described them as a system that could be used, not only as a delivery system, but also as adjuvants [15,46,47]. Designing the protein-based liposomal adjuvant vaccine could be an approach to attaining a maximum efficacy and low toxicity [48]. Similarly, in this scenario, the liposomes had shown their non-toxic nature for the chosen HEp-2 cells for 72 h, which reflected their potential application in designing the RSV treatment. The cytotoxicity of the various concentrations of these liposomes was tested by the MTT assay, and it was found that the liposome formulations of two different concentrations of lipids, with and without protein loading, did not render any cytotoxic effect or molecular effect on the host HEp-2 cells (Figure 4).

Amongst the several ways of viral inhibition analysis, the plaque assay, immunofluorescence microscopy, and qPCR were considered as standard tools [13,21,49]. The anti-RSV effect was therefore confirmed in the plaque assay (Figure 5), immunofluorescence imaging (Figure 6), and qRT-PCR. The anti-RSV activity of the peptide RF-282 had already been reported, but an interesting finding confirmed the inhibition of the RSV in the presence of the DSPC cholesterol liposomes, which resembled the results from Hendricks et al., where the decoy liposomes were found to be capable of inhibiting RSV from cellular binding [23]. The functionalization of the gold nanoparticles using the anti-RSV peptide was shown to have a significant effect on the RSV inhibition, compared with the peptide alone [20,21]. Similarly, our results also confirmed that the RSV percent inhibition was significantly increased (*p* <0.05) for the RF-482 loaded liposomes compared with the RF-482 and liposomes alone (Figure 5 and Figure 7).

Overall, our research demonstrated that the RF-482 itself was capable of inhibiting RSV, but the inhibition of RSV was significantly increased with the loading on the liposome. Moreover, recent reports showed that phosphatidylinositol (PI) inhibited the respiratory syncytial virus (RSV), as the PI bound the RSV with a high affinity, which inhibited its fusion to the epithelial cells [31,50]. There were five derivatives of the phospholipids, including the PI and the phosphocholine (PC). Although the exact mechanism of RSV inhibition by PC was not confirmed, it was possible that, similarly to the PI, the PC could have an affinity towards the RSV, inhibiting its fusion to the epithelial cells. However, the liposomal loading of the anti-RSV agents, like RF-482, were shown to have decreased the viral activity of RSV. Liposomes are multifaceted delivery systems and are capable of co-encapsulating compounds depending on their characteristics [51]. This structural attribute of the liposomes could become a carrier of multiple proteins and/or other anti-RSV compounds. Liposomes could be designed to look like a virus, by attaching multiple proteins to it [52]. Therefore, liposomes are multifaceted systems and hold the potential of entering the mainstream for designing the prophylaxis against RSV infection.

## 5. Conclusions

RF-482 has been reported as an inhibitor of RSV fusion and, to date, gold nanoparticles are the only reported carrier of RF-482 [21]. However, liposomes have been considered as an alternative carrier for delivering the anti-RSV peptide, RF-482. Moreover, the liposomes, as a new candidate for RSV inhibition, have been tested and the inhibitory effect of the liposomes has been shown to be better compared with the peptide alone, while the peptide-loaded liposomes have proved to be a better candidate for the RSV inhibition compared with both the peptide and liposomes alone. However, this has generated another quest to unveil the exact mechanism of the RSV infection inhibition, using the liposomes or peptide-loaded liposomes, which could lead to the commercialization of the formulation.
